# The relative importance of factors predicting outcome for myeloma patients at different ages: results from 3894 patients in the Myeloma XI trial

**DOI:** 10.1038/s41375-019-0595-5

**Published:** 2019-10-14

**Authors:** Charlotte Pawlyn, David Cairns, Martin Kaiser, Alina Striha, John Jones, Vallari Shah, Matthew Jenner, Mark Drayson, Roger Owen, Walter Gregory, Gordon Cook, Gareth Morgan, Graham Jackson, Faith Davies

**Affiliations:** 1 The Institute of Cancer Research, London, UK; 2 The Royal Marsden Hospital NHS Foundation Trust, London, UK; 3 Clinical Trials Research Unit, Leeds Institute of Clinical Trials Research, University of Leeds, Leeds, UK; 4 University Hospital Southampton NHS Foundation Trust, Southampton, UK; 5 Clinical Immunology Service, Institute of Immunology and Immunotherapy, University of Birmingham, Birmingham, UK; 6 Haematological Malignancy Diagnostic Service (HMDS), St James’s University Hospital, Leeds, UK; 7 Section of Experimental Haematology, Leeds Institute of Cancer and Pathology, University of Leeds, Leeds, UK; 8 Perlmutter Cancer Center, NYU Langone Health, New York, UK; 9 Northern Institute for Cancer Research, Newcastle University, Newcastle upon Tyne, UK

**Keywords:** Translational research, Myeloma, Genetics research

## Abstract

Disease factors such as tumor burden and molecular risk affect myeloma patient outcomes as well as patient factors that impact the capacity to deliver treatment. How the relative importance of these factors changes with patient age has not previously been investigated comprehensively. We analyzed data from 3894 patients of all ages uniformly treated in a large clinical trial of myeloma patients, Myeloma XI. Even with novel therapeutic approaches progression-free survival (PFS) and overall survival (OS) are affected by age with a stepwise reduction in PFS and OS with each decade increase. Renal function deteriorated with increasing age whilst the frequency of *t*(4;14) and del(17p) decreased and gain(1q) increased. The relative contribution of performance status, international staging score and molecular risk to progression-free and overall survival varied by age group. Molecular events have a larger effect on outcome in younger patients with their relative contribution diminishing in the elderly. Performance status is important for patient outcome at all ages suggesting that physical frailty may be a more important predictor of outcome than age itself. Significant differences in the factors driving patient outcomes at different ages are important to consider as we design disease segmentation strategies to deliver personalized treatment approaches.

## Background

Myeloma patient outcomes have improved in the last two decades following the introduction of proteasome inhibitor and immunomodulatory drug therapies. However, there remains a subset of patients with high-risk disease who continue to have poor outcomes [1, 2]. Phenotypically, high-risk myeloma is characterized by early progression and death. Factors contributing to poor outcomes include patient-related variables, such as comorbidities and organ reserve limiting treatment delivery [3], and tumor-related factors such as enhanced proliferation and apoptosis resistance driven by molecular driver lesions [1].

From a molecular point of view there is not one single lesion universally associated with high-risk disease but rather this is due to the interplay of a range of different molecular abnormalities. Chromosomal abnormalities typical of myeloma include the tumor initiating lesions hyperdiploidy and translocations involving the immunoglobulin regions and lesions associated with disease progression, such as copy number change [4–6]. Translocations *t*(4;14), *t*(14;16) and *t*(14;20) together with the copy number abnormalities del(17p), gain/amp(1q) have all been associated with adverse outcomes and the presence of more than one adverse lesion is associated with even worse prognosis [7–9]. Mutations have also been associated with poor outcomes with biallelic inactivation of *TP53* by mutation and/or deletion associated with the worst outcomes [10–12].

Most molecular studies to date have been conducted with a focus on younger patients. In older patients it has become increasingly apparent that outcomes are nuanced by physical functioning and comorbidities, which play a role in treatment tolerability and the dose intensity delivered over time. This suggests that not all factors predictive of outcome will have equal significance at all ages. Unfortunately, recruitment of older patients to clinical trials is often difficult and few trials have sufficient power to examine the true impact of different variables in patients over the age of 75 years [13, 14].

In this work we determined the relative contribution of patient and disease factors on clinical outcome and how these are impacted by age in a uniformly treated trial population in the UK NCRI Myeloma XI study.

## Methods

Myeloma XI is a multicenter, phase III, open-label, randomized controlled trial for newly diagnosed myeloma patients, with pathways for transplant eligible and non-eligible patients. The trial recruited 3894 patients from both academic and district general hospitals around the UK between 2010 and 2016. The study was approved by the national ethics review board (National Research Ethics Service, London, UK), institutional review boards of the participating centers, and the competent regulatory authority (Medicines and Healthcare Products Regulatory Agency, London, UK), and was undertaken according to the Declaration of Helsinki and the principles of Good Clinical Practice as espoused in the Medicines for Human Use (Clinical Trials) Regulations. Informed consent was obtained from all patients. Some of the primary study outcomes have been reported elsewhere [15]. The study was designed to be representative of the general myeloma population in the UK with only a few exclusion criteria. Eligible patients for the overall study were aged ≥18 years and had newly diagnosed MM, based on paraprotein in serum and/or urine, bone marrow clonal plasma cells or plasmacytoma, and myeloma-related symptoms or organ or tissue impairment. Patients were excluded if they had other previous or concurrent malignancies, including myelodysplastic syndromes; prior treatment for myeloma (excluding local radiotherapy, bisphosphonates, and corticosteroids); grade ≥ 2 peripheral neuropathy; acute renal failure (unresponsive to up to 72 h of rehydration, characterized by creatinine level > 500 µmol/L or urine output < 400 mL/day, or requiring dialysis); or active or prior hepatitis C infection. The study is registered with the ISRCTN registry, number ISRCTN49407852, and clinicaltrialsregister.eu, number 2009-010956-93, and has completed recruitment.

In brief, the trial compared a triplet combination of cyclophosphamide, lenalidomide, and dexamethasone to a similar combination with thalidomide (CRD vs. CTD). Patients were treated for a minimum of four (if transplant eligible) or six (if transplant non-eligible) cycles and to maximum response. For patients with a suboptimal response defined as <VGPR there was a subsequent randomization to a proteasome inhibitor containing triplet (cyclophosphamide, bortezomib, and dexamethasone, CVD) or no further therapy. All patients with stable disease or progression at the end of initial induction received CVD. Transplant eligible patients received an autologous stem cell transplant with melphalan induction. All patients underwent a maintenance randomization, which compared lenalidomide maintenance (±vorinostat) till disease progression vs. observation.

Baseline variables were collected for all patients. Adverse molecular lesions were determined by qRT-PCR and MLPA in a central laboratory and were available for 1567/3894 patients. In this approach qRT-PCR is used to assay the expression of translocation gene partners including *t*(4;14): *MMSET*, *FGFR3*; *t*(14;16): *MAF* and *t*(14;20) *MAFB*. MLPA was used to assay copy number by including probesets at sites of the commonly deleted and amplified regions in myeloma e.g., at genes *CKS1B* on 1q21.3 and *TP53* on 17p13. These techniques have been previously validated and provide equivalent results to interphase fluorescence in situ hybridization (iFISH) commonly used in clinical practice [16, 17]. Adverse lesions were defined as *t*(4;14), *t*(14;16), *t*(14;20), del(17p) and gain(1q) based on our previous study [7]. Standard risk (SR) was defined as the absence of any of these lesions, high-risk (HiR) one lesion and ultra high-risk (UHiR) >1 lesion.

In this analysis we included all patients enrolled into the Myeloma XI study and summarized important baseline variables by age in decade groups <60 years (yr), 61–70 yr, 71–80 yr and >80 yr. Variables were compared using Fisher’s Exact test for categorical characteristics and the Wilcoxon–Mann–Whitney test for continuous characteristics with *P* < 0.05 the level considered statistically significant. A multivariate Cox regression analysis was performed within each age group to identify the variables with the greatest effect on outcome using complete-case data. The explained variation in time-to-event endpoints was quantified using ${R_{\rm{D}}^{2}}$ [18] and as a proportion of ${R_{\rm{D}}^{2}}$ for the chosen “best” model. Relative survival estimates were obtained using flexible parametric survival models on the hazard scale with four degrees of freedom [19]. Relative survival was defined as the observed survival divided by the expected survival where the expected survival is obtained from national life tables stratified by age at diagnosis, sex and calendar year. United Kingdom life-time risk was estimated from data available from the Office for National Statistics (https://www.ons.gov.uk/peoplepopulationandcommunity/birthsdeathsandmarriages/lifeexpectancies/datasets/nationallifetablesunitedkingdomreferencetables). Statistical analysis was performed using SAS v9.4 (SAS Institute Inc., Cary, NC) and Stata IC v13 (StataCorp. College Station, TX: StataCorp LLC). Some graphs were drawn using Prism (v7 Graphpad Software Inc.).

## Results

### The effect of age on primary trial outcomes PFS and OS

**Fig. 1:**
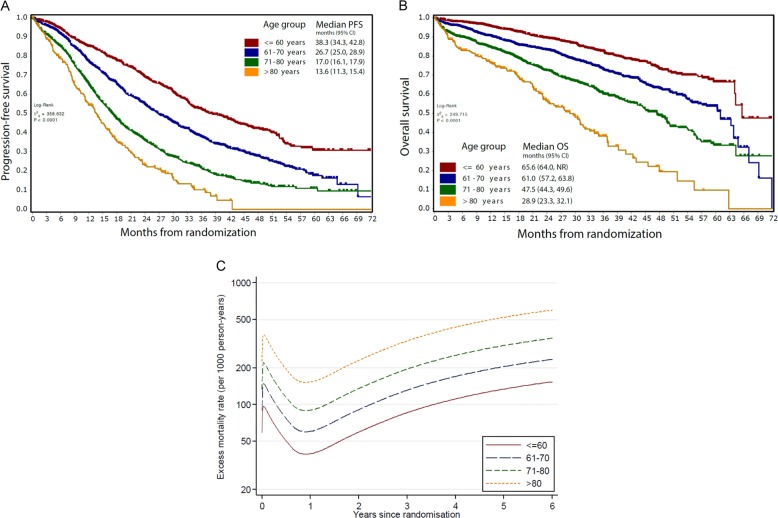
Kaplan–Meier survival curves by age group. **a** Progression free survival **b** Overall survival

The median age of all patients enrolled in the Myeloma XI study was 68 years (range 28–92) and their other baseline characteristics and treatment are shown in Table 1. A significant proportion of patients were aged over 80 years (≤60 yr (*n* = 982), 61–70 yr (*n* = 1418), 71–80 yr (*n* = 1247) and >80 yr (*n* = 247)). Age was strongly predictive of both progression-free (PFS) and overall survival (OS) (Fig. 1a, b). Patients over the age of 80 yr have particularly poor outcomes with median PFS 13.6 months (95% CI [11.3, 15.4]) and OS 28.9 months (95% CI [23.3, 32.1]) compared with 38.3 [34.3, 42.8] and 65.6 [64.0, NR] respectively for patients aged under 60 yr. For each decade increase in age there was a significant stepwise reduction in PFS and OS with no overlap in 95% confidence intervals between any adjacent groups (Fig. 1a, b). A comparison of overall survival using estimates of United Kingdom life-time risk from data available from the Office for National Statistics confirmed an increase in excess deaths with increasing age of myeloma patients (Fig. 1c and Supplementary Fig. 1) compared with the general population.

**Table 1:** Baseline characteristics of the Myeloma XI population

Characteristic	Number of patients (%) total *n* = 3894
Sex	
Male	2268 (58.2%)
Female	1626 (41.8%)
Age at initial randomization	
Mean (SD)	66.6 (10.23)
Median (range)	68 (28, 92)
WHO performance status	
0	1338 (34.4%)
1	1540 (39.5%)
2	596 (15.3%)
3	187 (4.8%)
4	21 (0.5%)
Missing	212 (5.4%)
Paraprotein type	
IgG	2394 (61.5%)
IgA	953 (24.5%)
IgM	14 (0.4%)
IgD	32 (0.8%)
Light chain only	454 (11.7%)
Non-secretor	24 (0.6%)
Missing	23 (0.6%)
Light chain type	
Lamba	1282 (32.9%)
Kappa	2552 (65.5%)
Missing	60 (1.5%)
Randomized induction treatment	
CTD	1021 (26.2%)
CRD	1021 (26.2%)
CTDa	924 (23.7%)
CRDa	928 (23.8%)
Maintenance treatment	
No maintenance	694 (17.8%)
Lenalidomide ± vorinostat	1164 (29.9%)
Did not undergo maintenance randomization	2036 (52.3%)

*SD* standard deviation, *CTD* cyclophosphamide, thalidomide and dexamethasone, *CRD* cyclophosphamide, lenalidomide and dexamethasone, *CTDa* attenuated CTD in the transplant non-eligible pathway, *CRDa* attenuated CRD in the transplant non-eligible pathway

We sought to explain these differences in PFS and OS by examining patient-related, laboratory and risk variables across the age groups.

### The effect of age on baseline variables

**Fig. 2:**
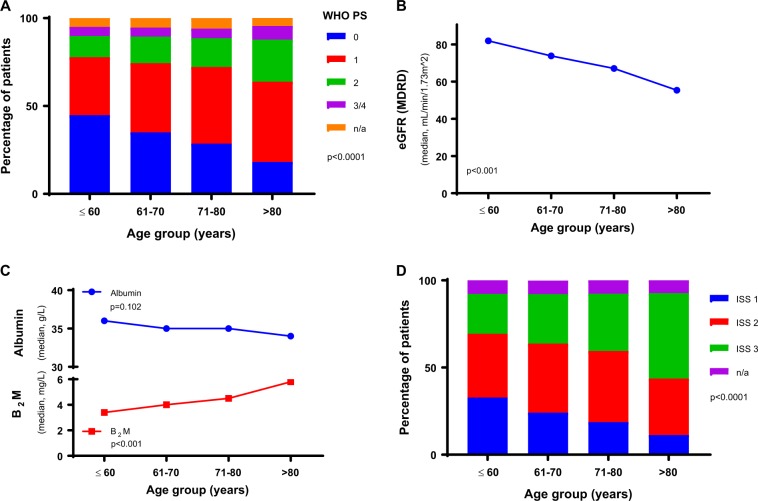
Baseline patient characteristics and laboratory parameters by age group. **a** Distribution of WHO performance status by age group. **b** Median eGFR by MDRD values indicative of renal impairment by age group. **c** Median values of B_2_M and albumin by age group. **d** Distribution of ISS by age group. In all graphs *p* values indicate an assessment of difference between the age groups (Fisher’s Exact test for categorical variables and the Wilcoxon–Mann–Whitney test for continuous variables). NS = not significant. n/a = not available. WHO PS = World Health Organization Performance Status. eGFR (MDRD) = estimated glomerular filtration rate by Modification of Diet in Renal Disease Study equation. ISS = International Staging Score

There was an excess of males compared with females across the trial with no significant difference in the proportions between age groups (Supplementary Fig. 2A) (Female % ≤60 yr 41.3%, 61–70 yr 40.2%, 71–80 yr 43.8% and >80 yr 42.1% *p* = NS). WHO performance status deteriorated significantly with advancing age (Fig. 2a), the proportion of patients with performance status 0–1 reduced from 81.9% for age ≤60 years to 67.0% for those aged >80 years (*p* < 0.0001). WHO performance status, however, retained prognostic impact in all age groups (Supplementary Fig. 3). There was a significant increase in the time from when patients first presented with signs/symptoms at the hospital to date of randomization (Supplementary Fig. 2B) with advancing age.

The proportion of patients with a hemoglobin less than the predefined cut off for sex altered between age groups (Supplementary Fig. 2C). There was an age related decline in renal function (Fig. 2b), as seen in our previous study MRC Myeloma IX, that was not related to a higher prevalence of high serum free light chain levels in the older age groups [20]. Instead there were fewer cases of light chain only myeloma in the older cohorts (Supplementary Fig. 3D). No single paraprotein type increased with age. Calcium did not change across age groups (Supplementary Fig. 2E). B_2_M significantly increased with age (Fig. 2c), which may be a reflection of the renal function deterioration. Albumin decreased with age (Fig. 2c) although this did not reach statistical significance, however in combination with the B_2_M increase there was an increase in the proportion of patients with higher International Staging System (ISS) stages in the older groups (Fig. 2d). ISS retained prognostic impact in all age groups (Supplementary Fig. 4).

**Fig. 3:**
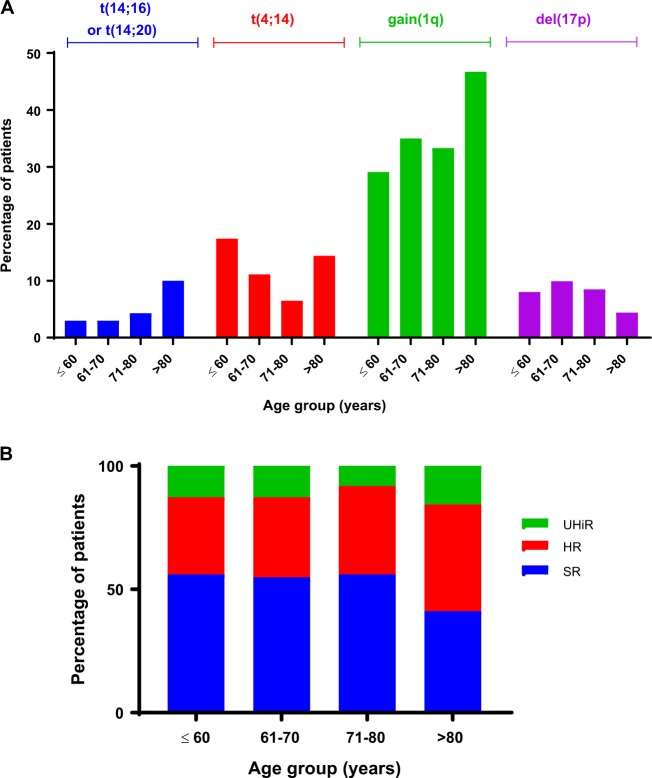
Molecular risk parameters at baseline by age group. **a** Adverse translocations and adverse copy number abnormalities. **b** Distribution of molecular risk group by age group. SR = standard risk, HiR = high risk, UHiR = Ultra-high risk. High-risk molecular abnormalities were defined as gain(1q), *t*(4;14), *t*(14;16), *t*(14;20), and del(17p). Ultra-high risk was defined as the presence of more than one high-risk lesion

Molecular data was available for 1567 patients. The proportion of patients with *t*(4;14) and del(17p) (Fig. 3a) fell significantly with age whilst those with gain(1q) increased. Overall the proportion of patients with high or ultra-high risk molecular abnormalities was broadly consistent across the lower three age groups (Fig. 3b). In the over 80s there was a slightly higher proportion of patients classified as high or ultra-high risk.

### The relative importance of tumor and patient variables by age

**Fig. 4:**
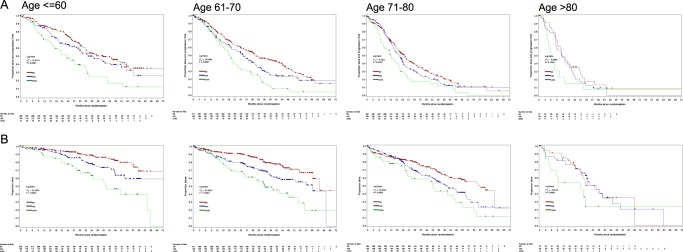
Kaplan–Meier survival curves by molecular risk group within each age group. **a** Progression free survival **b** Overall survival. SR = standard risk (red), HiR = high risk (blue), UHiR = Ultra-high risk (green). High-risk molecular abnormalities were defined as gain(1q), *t*(4;14), *t*(14;16), *t*(14;20), and del(17p). Ultra-high risk was defined as the presence of more than one high-risk lesion

We have previously demonstrated that molecular risk lesions predict outcome with groups defined as standard, high, and ultra high-risk [9]. We confirmed that these groups remain predictive for both PFS (Fig. 4a) and OS (Fig. 4b) within each age group but show that the degree of separation between the risk categories lessened with advancing age. In the over 80 year group the presence of only one lesion alone does not associate with adverse outcome. The presence of del(17p) retained an important effect on outcome at all ages whilst the adverse effect of *t*(4;14) and gain(1q) was reduced in the very elderly (Table 2).

**Table 2:** Effect on outcome of individual molecular risk lesions

Age group	≤60 (*n* = 402)		61–70 (*n* = 568)		71–80 (*n* = 507)		>80 (*n* = 90)	
	HR [95% CI]	*P* value	HR [95% CI]	*P* value	HR [95% CI]	*P* value	HR [95% CI]	*P* value
PFS								
*t*(4;14)	1.79 [1.19, 2.70]	**0.0047**	1.67 [1.22, 2.27]	**0.0012**	1.59 [1.05, 2.38]	**0.0272**	1.35 [0.71, 2.56]	0.3601
del(17p)	1.35 [0.81, 2.22]	0.2442	1.67 [1.19, 2.27]	**0.0016**	1.61 [0.19, 2.27]	**0.0063**	3.45 [1.25, 10.0]	**0.0107**
gain(1q)	1.39 [0.98, 1.96]	0.06	1.39 [1.11,1.72]	**0.0035**	1.18 [0.95,1.47]	0.1318	1.25 [0.79, 1.96]	0.3399
OS								
*t*(4;14)	1.96 [1.11, 3.45]	**0.0189**	1.92 [1.25, 2.86]	**0.002**	1.16 [0.66, 2.04]	0.5903	1.08 [0.48, 2.38]	0.8693
del(17p)	2.86 [1.56, 5.26]	**0.0004**	2.63 [1.75, 4.00]	**<0.0001**	2.22 [1.43, 3.57]	**0.0003**	4.00 [1.43, 11.1]	**0.0043**
gain(1q)	1.85 [1.11, 3.03]	**0.0163**	1.67 [1.21, 2.27]	**0.0016**	1.52 [1.14, 2.04]	**0.0046**	1.05 [0.60, 1.85]	0.8534

Hazard ratio and *P* value for presence vs. absence of each risk lesion. (*P* values in bold if <0.05)

**Fig. 5:**
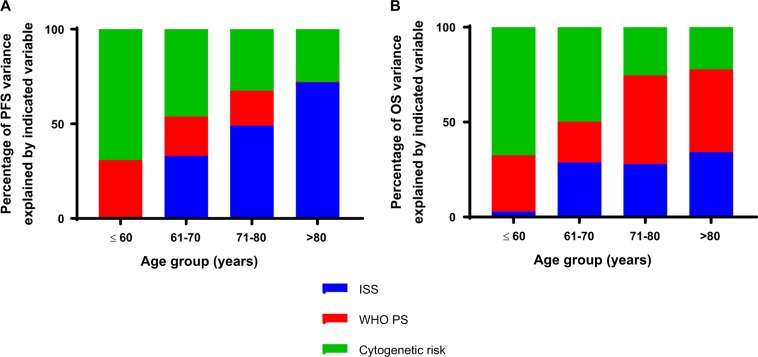
The percentage of variance explained by molecular risk, ISS, and WHO PS. **a** Progression free survival **b** Overall survival. The effect of age (PFS: *P* < 0.0001; OS: *P* < 0.0001), performance status (PFS: *P* = 0.0001; OS: *P* < 0.0001), ISS (PFS: *P* < 0.0001; OS: *P* < 0.0001) and molecular risk (PFS: *P* < 0.0001; OS: *P* < 0.0001) on clinical outcomes is statistically significant. SR = standard risk, HiR = high risk, UHiR = Ultra-high risk. High-risk moelcular abnormalities were defined as gain(1q), *t*(4;14), *t*(14;16), *t*(14;20), and del(17p). Ultra-high risk was defined as the presence of more than one high-risk lesion. ISS = International Staging Score. WHO PS = World Health Organization Performance Status

We investigated the relative importance of molecular events compared with patient related variables by age group in standard and relative survival models. Multivariate analysis of both PFS and OS using a Cox regression model was performed within each age group. The following variables with the greatest effect on outcome were included in the “best” model; WHO performance status (PS, 0–4), International staging system (ISS, I–III) and molecular risk (UHiR, HiR, and SR). The percentage of variation explained by each of these variables was compared between age groups (Fig. 5). With advancing age molecular risk had a smaller influence on outcome, and ISS a greater influence, compared with other factors. Performance status had a clear impact on outcome at all ages suggesting that physical frailty might be a more important determinant of outcome than age itself. The patterns were the similar for PFS and OS.

## Discussion

For the first time we have demonstrated the relative importance of myeloma patient and disease-specific variables at different ages and their effect on patient outcome. Whilst factors including ISS and cytogenetic risk remain prognostic at all ages, we present data showing that their relative importance alters. We found an increasing influence of ISS on outcome with increasing age and a diminishing influence of molecular risk status. Performance status retains prognostic power at all ages suggesting that physical frailty may be a more important predictor of outcome than age itself. Although ISS may be considered a disease-related score, there is likely to be a significant impact on ISS driven by patient factors and a patient’s ability to tolerate their disease burden. For example high levels of B_2_M are associated with renal impairment, and we demonstrate a reduction in eGFR with age, whilst albumin tends to fall with increasing age. These patient factors may therefore be influencing ISS stage and its impact on outcome.

Importantly we identify a stark difference in outcomes for myeloma patients of different ages, even with regimens including novel therapies. When outcomes are normalized to the UK population we show that patients aged over 80 can expect a median progression-free and overall survival of only 40% that for patients aged <60.

In our previous study, in the era prior to novel agents, (published in 2005) we demonstrated a reduction in the frequency of IgH translocations with age and no prognostic effect of *t*(4;14) and del(17p) on outcome for patients over 70 [21]. Findings from that study were limited by small numbers of patients as well as having limited applicability to patients treated with current standards of care. More recently, an analysis of Intergroupe Francophone Myeloma demonstrated that the *t*(4;14) and del(17p) retained prognostic significance in patients over 65 years but they also noted that these lesions had a lower frequency compared with younger patients [22]. In the Myeloma XI dataset we have a much larger number of patients and one of the strengths of the trial is that it had very limited exclusion criteria making it much more generalizable to ‘real-world’ situations whilst retaining the rigor of a phase III study.

We show that care needs to be taken in risk assignment using the common myeloma risk scores. The Myeloma Genome Project Double Hit Classification [12] and Mayo Clinic mSMART criteria [23] rely heavily on tumor factors rather than patient factors. The Double Hit analysis excluded patients over the age of 75 because of the significant impact of this factor on outcome. Our findings suggest these scores may be very appropriate for younger patients but, whilst they may retain prognostic significance in older patients, different outcome prediction tools may be better suited for these groups. We have recently developed a risk profiling approach for transplant-ineligible older patients [24], and other similar approaches based on assessment of frailty have also been proposed [25–27].

The R-ISS [8] risk score incorporates cytogenetics together with the ISS and LDH but does not incorporate performance status and a criticism of our analysis could be that we did not include this score in our multivariate analysis. This was not done mainly because by keeping the ISS and molecular risk factors separate the contribution of each to outcome at different ages could be assessed. Further we only had a limited proportion of patients with LDH data available. In addition, a study of the relative contribution of patient frailty scores to outcome at different ages would have been beneficial but these were not performed within the Myeloma XI study. Ultimately scoring systems that incorporate a wide range of factors, perhaps weighted differently based on age, could provide a unified solution.

We have demonstrated that the spectrum and relative importance of patient-specific and tumor acquired biological and genetic features changes significantly across age-groups of myeloma patients. This is important to understand as we design disease segmentation strategies to deliver personalized treatment approaches aiming to improve patient outcomes. The focus of such approaches in younger patients should be to target the biology underlying high-risk disease, whilst in older patients the strategy will require a focus on clinical variables and treatment type and intensity modification.

## Supplementary information

Supplementary Information
