# Supplementary material for: The relative importance of factors predicting outcome for myeloma patients at different ages: results from 3894 patients in the Myeloma XI trial
Source: Leukemia. 2019 Oct 14;34(2):604–12. doi: 10.1038/s41375-019-0595-5 (PMC7214257; doi:10.1038/s41375-019-0595-5)
Supplement: Supplementary file 1 — Supplementary Information [file 41375_2019_595_MOESM1_ESM.pdf]

The relative importance of factors predicting outcome for myeloma patients at different ages: Results from 3894 patients in the Myeloma XI trial.

Charlotte Pawlyn[1,2*], David Cairns[3], Martin Kaiser[1,2], Alina Striha[3], John Jones[1], Vallari Shah[1], Matthew Jenner[4], Mark Drayson[5], Roger Owen[6], Walter Gregory[3], Gordon Cook[7], Gareth Morgan[8], Graham Jackson[9], Faith Davies[8]

1. The Institute of Cancer Research, London, UK
2. The Royal Marsden Hospital NHS Foundation Trust, London, UK
3. Clinical Trials Research Unit, Leeds Institute of Clinical Trials Research, University of Leeds, Leeds
4. University Hospital Southampton NHS Foundation Trust, Southampton
5. Clinical Immunology Service, Institute of Immunology and Immunotherapy, University of Birmingham, Birmingham, UK
6. Haematological Malignancy Diagnostic Service (HMDS), St James's University Hospital, Leeds, UK
7. Section of Experimental Haematology, Leeds Institute of Cancer and Pathology, University of Leeds, Leeds, UK
8. Myeloma Center, University of Arkansas for Medical Sciences, Little Rock, USA
9. Northern Institute for Cancer Research, Newcastle University, Newcastle upon Tyne, UK

Supplementary Data Contents

Supplementary Figure 1- Difference in excess mortality rates compared to matched UK nationals 2

Supplementary Figure 2- Patient characteristics and laboratory parameters at baseline by age group 3

Supplementary Figure 3- Outcomes by WHO Performance status 5

Supplementary Figure 4 - Outcomes by International Staging System 7

Supplementary Figure 1- Difference in excess mortality rates compared to matched UK nationals

Comparison between age groups

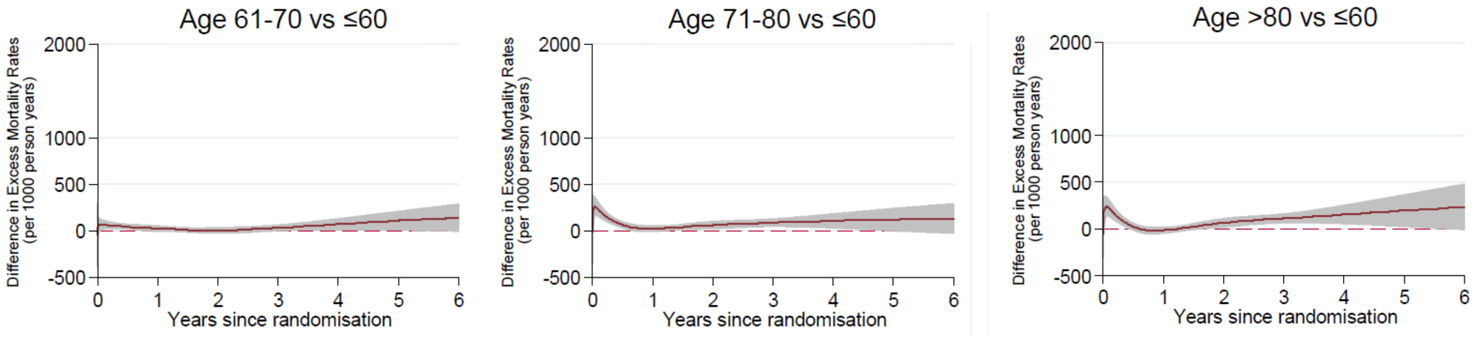

Supplementary Figure 2- Patient characteristics and laboratory parameters at baseline by age group

A) Number of patients within each age group by sex. B) Delay between presentation and randomization by age group. C) Percentage of patients with anemia by age group. D) Distribution of disease secretion subtype by age group. E) Percentage of patients with hypercalcemia by age group. F) Percentage of patients with bone marrow plasma cells ≥20% by age group. In all graphs p values indicate an assessment of difference between the age groups (Fisher's Exact test for categorical characteristics and the Wilcoxon-Mann-Whitney test for continuous characteristics). NS = not significant. n/a = not available.

A

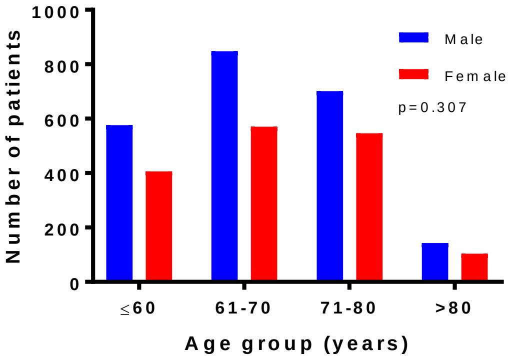

B

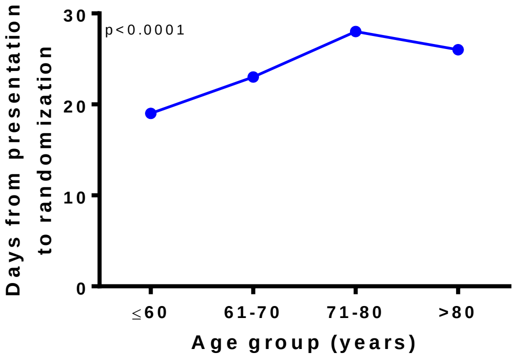

C

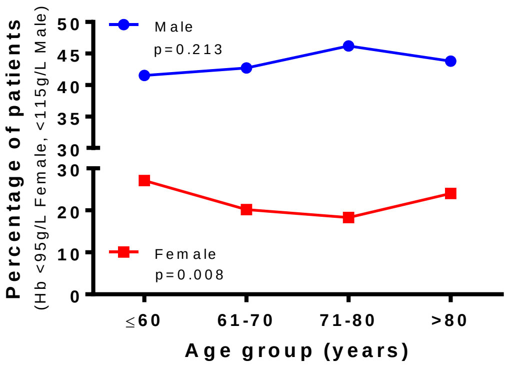

D

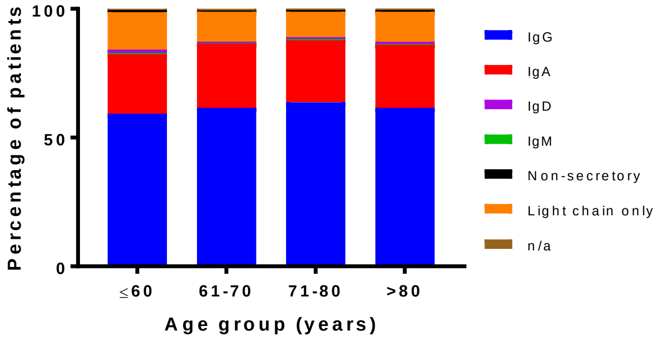

E

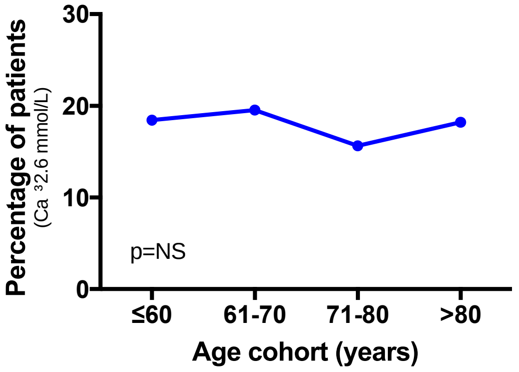
Percentage of patients
(Ca ³2.6 mmol/L)
30
20
10
0
p=NS
≤60
61-70
71-80
>80
Age cohort (years)

F

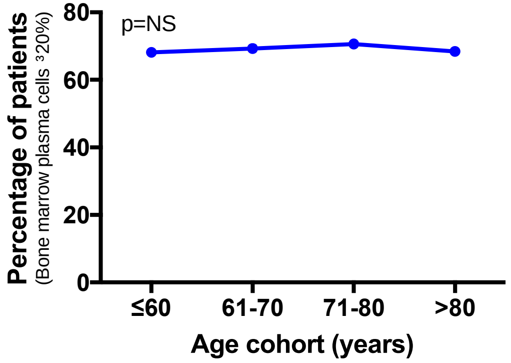
Percentage of patients
(Bone marrow plasma cells ³20%)
80
60
40
20
0
p=NS
≤60
61-70
71-80
>80
Age cohort (years)

Supplementary Figure 3- Outcomes by WHO Performance status

Kaplan-Meier survival curves by WHO Performance status (PS) within each age group. A) Progression free survival B) Overall Survival.

A)

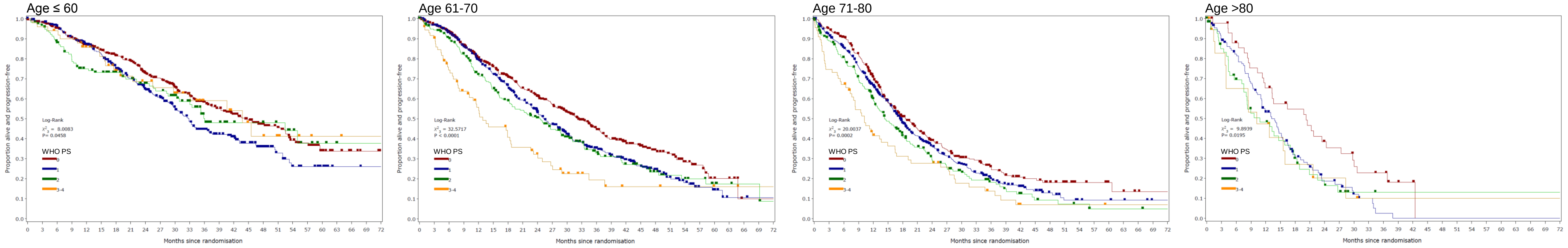

Age ≤ 60
Log-Rank
χ^2_3 = 8.0083
P= 0.0458
WHO PS
0
1
2
3-4
Proportion alive and progression-free
Months since randomisation
Age 61-70
Log-Rank
χ^2_3 = 32.5717
P < 0.0001
WHO PS
0
1
2
3-4
Proportion alive and progression-free
Months since randomisation
Age 71-80
Log-Rank
χ^2_3 = 20.0037
P= 0.0002
WHO PS
0
1
2
3-4
Proportion alive and progression-free
Months since randomisation
Age >80
Log-Rank
χ^2_3 = 9.8939
P= 0.0195
WHO PS
0
1
2
3-4
Proportion alive and progression-free
Months since randomisation

B)

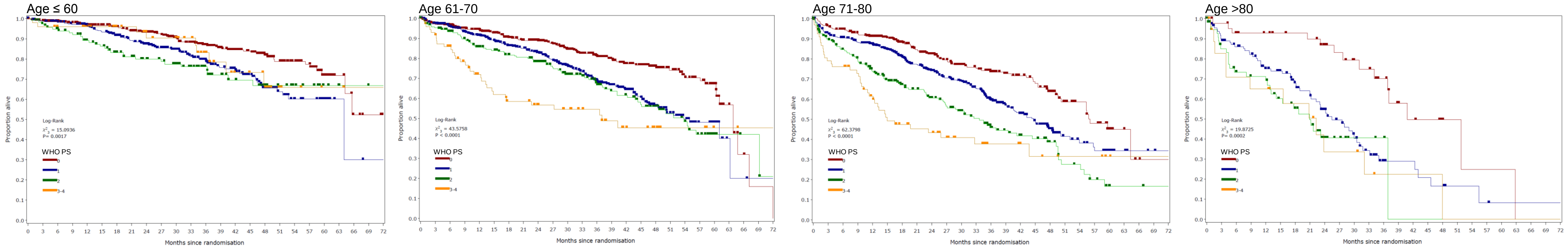

Age ≤ 60
Log-Rank
χ^2_3 = 15.0936
P= 0.0017
WHO PS
0
1
2
3-4
Proportion alive
Months since randomisation
Age 61-70
Log-Rank
χ^2_3 = 43.5758
P < 0.0001
WHO PS
0
1
2
3-4
Proportion alive
Months since randomisation
Age 71-80
Log-Rank
χ^2_3 = 62.3798
P < 0.0001
WHO PS
0
1
2
3-4
Proportion alive
Months since randomisation
Age >80
Log-Rank
χ^2_3 = 19.8725
P= 0.0002
WHO PS
0
1
2
3-4
Proportion alive
Months since randomisation

Supplementary Figure 4 - Outcomes by International Staging System

Kaplan-Meier survival curves by International Staging System (ISS) within each age group. A) Progression free survival B) Overall Survival.

A)

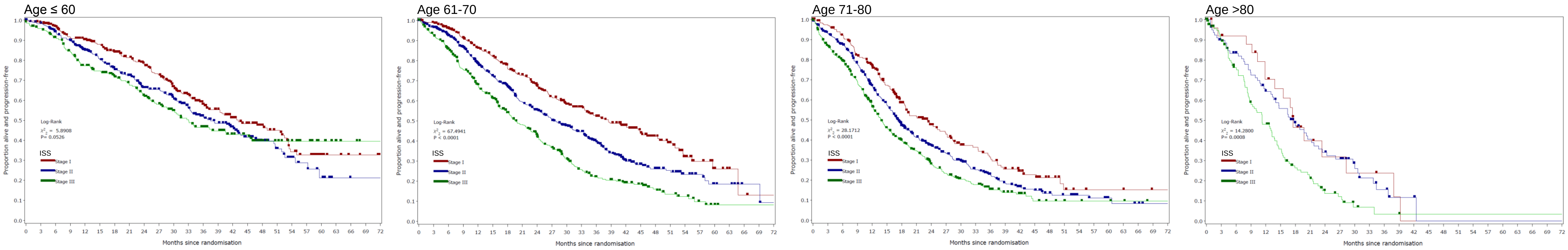

Age ≤ 60
Age 61-70
Age 71-80
Age >80
Proportion alive and progression-free
Months since randomisation
Log-Rank
χ^2_2 = 5.8908
P= 0.0526
χ^2_2 = 67.4941
P < 0.0001
χ^2_2 = 28.1712
P < 0.0001
χ^2_2 = 14.2800
P= 0.0008
ISS
Stage I
Stage II
Stage III

B)

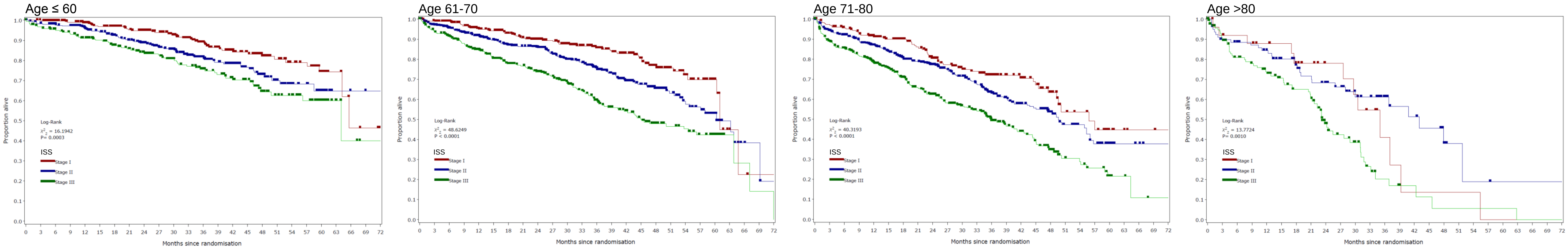

Age ≤ 60
Age 61-70
Age 71-80
Age >80
Proportion alive
Months since randomisation
Log-Rank
χ^2_2 = 16.1942
P= 0.0003
χ^2_2 = 48.6249
P < 0.0001
χ^2_2 = 40.3193
P < 0.0001
χ^2_2 = 13.7724
P= 0.0010
ISS
Stage I
Stage II
Stage III
